# PASCAL: a pseudo cascade learning framework for breast cancer treatment entity normalization in Chinese clinical text

**DOI:** 10.1186/s12911-020-01216-9

**Published:** 2020-08-28

**Authors:** Yang An, Jianlin Wang, Liang Zhang, Hanyu Zhao, Zhan Gao, Haitao Huang, Zhenguang Du, Zengtao Jiao, Jun Yan, Xiaopeng Wei, Bo Jin

**Affiliations:** 1 School of Computer Science and Technology, Dalian University of Technology, No.2 Linggong Road, Ganjingzi District, Dalian, Liaoning, 116024 China; 2 First Hospital of Lanzhou University, 1 Donggang W Rd, Chengguan District, Lanzhou, Gansu, 730000 China; 3 International Bussiness College, Dongbei University of Finance and Economics, No.20 Jianshan Street, Shahekou District, Dalian, Liaoning, 116025 China; 4 Dalian University, No.10 Xuefu Street, Economic and Technological Development Zone, Dalian, Liaoning, 116622 China; 5 BeiJing Haoyisheng Cloud Hospital Management Technology Ltd., No.10 Dewai Street, Xicheng District, Beijing, 100088 China; 6 The People’s Hospital of Liaoning Province, No.33 Shenhe District, Shenyang, Liaoning, 110016 China; 7 AI Lab, Yidu Cloud, No.35 of Huayuan North Road, Haidian District, Beijing, 100191 China; 8 School of Innovation and Entrepreneurship, Dalian University of Technology, No.2 Linggong Road, Ganjingzi District, Dalian, Liaoning, 116024 China

**Keywords:** Breast cancer, Cascade learning, Treatment entity normalization, Chinese clinical text mining

## Abstract

**Backgrounds:**

Knowledge discovery from breast cancer treatment records has promoted downstream clinical studies such as careflow mining and therapy analysis. However, the clinical treatment text from electronic health data might be recorded by different doctors under their hospital guidelines, making the final data rich in author- and domain-specific idiosyncrasies. Therefore, breast cancer treatment entity normalization becomes an essential task for the above downstream clinical studies. The latest studies have demonstrated the superiority of deep learning methods in named entity normalization tasks. Fundamentally, most existing approaches adopt pipeline implementations that treat it as an independent process after named entity recognition, which can propagate errors to later tasks. In addition, despite its importance in clinical and translational research, few studies directly deal with the normalization task in Chinese clinical text due to the complexity of composition forms.

**Methods:**

To address these issues, we propose PASCAL, an end-to-end and accurate framework for breast cancer treatment entity normalization (TEN). PASCAL leverages a gated convolutional neural network to obtain a representation vector that can capture contextual features and long-term dependencies. Additionally, it treats treatment entity recognition (TER) as an auxiliary task that can provide meaningful information to the primary TEN task and as a particular regularization to further optimize the shared parameters. Finally, by concatenating the context-aware vector and probabilistic distribution vector from TEN, we utilize the conditional random field layer (CRF) to model the normalization sequence and predict the TEN sequential results.

**Results:**

To evaluate the effectiveness of the proposed framework, we employ the three latest sequential models as baselines and build the model in single- and multitask on a real-world database. Experimental results show that our method achieves better accuracy and efficiency than state-of-the-art approaches.

**Conclusions:**

The effectiveness and efficiency of the presented pseudo cascade learning framework were validated for breast cancer treatment normalization in clinical text. We believe the predominant performance lies in its ability to extract valuable information from unstructured text data, which will significantly contribute to downstream tasks, such as treatment recommendations, breast cancer staging and careflow mining.

## Background

Breast cancer is one of the leading cancers with a high mortality rate. WHO reported that it is the second most common cause of cancer death in women [1]. In particular, developing countries are suffering from an increasing breast cancer epidemic with a growing number of younger women who are susceptible to cancer. Fortunately, the mortality rate caused by breast cancer has significantly decreased in recent years due to the increased emphasis on early detection and the development of more effective treatment [2]. Additionally, the widespread application of modern medical devices has accumulated large-scale electronic health record (EHR) data, especially historical breast cancer treatment records, which create a foundation for drug therapy analysis, regimen adjustment, and careflow mining [3]. Consequently, breast cancer patients can receive better healthcare and more accurate treatment.

Additionally, traditional machine learning methods and more advanced deep learning methods have deeply accelerated the process of discovering underlying patterns or structures in EHR data. For instance, in the treatment prediction field, Yadav et al. [4] proposed a framework that uses a decision tree and support vector machine algorithm to identify patients who need urgent chemotherapy. For breast cancer diagnosis, Wang et al.[5] developed a comprehensive diagnosis tool by mining heterogeneous EHR data, such as physical examination results, patient clinical backgrounds, histories and features of mammography images. For prognosis, [6] employed three different machine learning methods to predict breast cancer survivability, which can assist in providing reasonable treatment for patients. In summary, the application of machine learning methods has largely improved the quality of patient care and reduced the misdiagnosis rate for breast cancer.

**Fig. 1:**
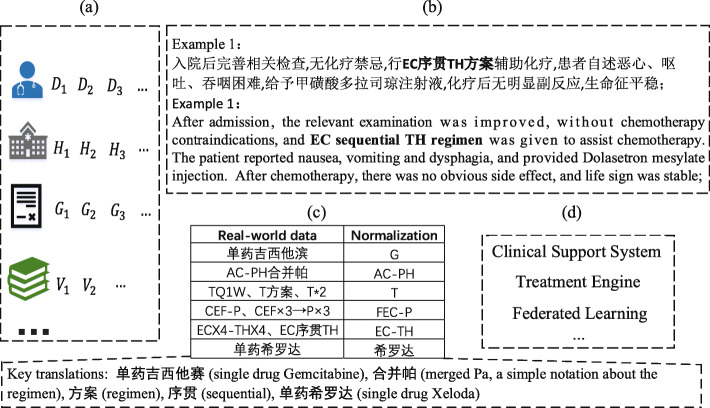
Illustration of clinical text, normalization examples and possible applications. **a** EHR data; **b** Clinical text from EHRs : an example of real clinical text and translated version; **c** Real-world data and standard entity; **d** Applied scenarios

Currently, most existing work on breast cancer treatment mining mainly relies on structural features or manually designed features based on EHRs in the English language. However, the widespread use of electronic medical devices in China has generated a considerable number of EHRs ranging from structured information to unstructured clinical text. As shown in Fig.1a, the EHR data might come from various hospitals and be recorded by different doctors under their own guidelines, thus making the final data rich in author- and domain-specific idiosyncrasies, acronyms and abbreviations. For instance, clinical physicians use “EC ×4-TH ×4” and “EC  TH” to denote the same treatment “EC-TH” (as shown in Fig. 1c). The complex character composition represents the specific treatment process in real clinical texts, which is helpful for future reference. In general, physicians use the fewest characters with the most powerful expressive ability in the treatment texts. Taking treatment “EC ×4-TH ×4” as an example, “ ×4” represents that the patient should adopt the EC as the first four chemotherapy regimens and employ the TH regimen as the subsequent four chemotherapy regimens.

However, such data hampers the development of advanced applications for breast cancer, such as treatment recommendation, treatment effect prediction, prognosis prediction and smart visualization in the era of big data. At present, uniform features have been utilized to avoid repetitive features and reduce noisy data, which can contribute to higher algorithm accuracy. For instance, standardized data have been used to solve the data isolated islands problem with the help of federated learning [7, 8]. Therefore, as shown in Fig. 1c, we need to normalize the medical terms in the left *real-world data* (Fig. 1b) to the right *normalized* term. Namely, despite various denotations for each treatment from the clinical text, according to the practical necessity, they must be mapped to a corresponding unified expression that generally comes from the authoritative reference such as *GUIDELINES* [9]. In our work, we call this a nontrivial problem (i.e., mapping the treatment entities to codes in a relevant controlled vocabulary) the treatment entity normalization task (TEN). Note that if the treatment entity is in the clinical text, we should first recognize the entity’s boundaries, which is called the treatment entity recognition task (TER). As shown in Fig. 1b, for the treatment “EC  TH  ” from the clinical text, we first recognize its position (TER task) and then map it to the unified term “EC-TH”.

At present, this is a challenging task for three reasons. First, the normalization process is tedious and time consuming via manual handling, thus requiring specifically designed data-driven approaches. Second, the medical entities are closely related to the contexts of clinical text, which provide a further description and should be taken into account when designing the algorithms, as shown in Fig. 1b. Finally, the inputs are mixed Chinese and English sentences (Fig. 1c), which make it more difficult to identify the entity boundaries. As a result, the development of computational methods concerning TEN has been hindered. In addition, researchers primarily focus on the named entity recognition task that determines the boundaries of medical entities, such as [10–13], while few studies directly deal with medical named entity normalization (MEN), especially for Chinese, due to the complexity of Chinese characters.

Nevertheless, researchers have proposed several methods, such as machine learning-based methods and joint learning-based methods, to address the named entity normalization problems. For example, Leaman et al. [14] were the first to introduce machine learning approaches to address the problem by pairwise learning. Leaman et al. [15] and Lou et al. [16] addressed these problems by jointly modeling recognition and normalization. Zhao et al. [17] proposed a deep neural multitask learning method with explicit feedback strategies to obtain optimal performance. However, all of the above methods are specifically designed for English-based entity normalization and recognition, such as from “CEF ×3-P ×3” to “FEC-P” in Fig. 1c. Chinese MEN is much more difficult than English owing to the complexity of Chinese composition forms and lack of word boundaries [18]. Moreover, the real-world public datasets in Chinese related to health informatics are almost nonexistent, which has been a bottleneck to the development of text mining algorithms in the Chinese domain. Additionally, in the Chinese medical named entity normalization domain, some researchers have developed algorithms by cooperating with hospitals. For instance, Luo et al. [19] introduced a multiview convolutional neural network to address the normalization of diagnostic and procedure names simultaneously. Likewise, Zhang et al. [20] presented an unsupervised framework to normalize the Chinese medical concept by combining disease text with comorbidity. However, the inputs of the networks are just Chinese medical terms, such as various name expressions for the same disease, not informative clinical sentences.

Furthermore, with the increasing quantity of training data, some researchers have begun to seek efficient learning algorithms, especially in the industrial field, such as [21]. In language modeling, many researchers [22, 23] attempt to leverage convolutional neural networks to replace traditional recurrent neural networks, which enable parallelization over the elements of sequences. Such approaches significantly promote computational efficiency compared with BiLSTM [24], which requires sequential modeling. In addition, to further improve the language model performance, Shen et al. [25] integrated a novel recurrent architecture with an explicit bias towards modeling a hierarchy of constituents, which can better extract the hidden hierarchical information in the sentence. In addition, with the advancement of health informatics research, the practical significance is becoming much more important, and it has brought about the necessity for computational efficiency. Therefore, we should maintain a balance between the computational precision and efficiency when developing such a framework.

To address the aforementioned challenges, we propose a pseudocascade learning framework (PASCAL) with a gated convolutional neural network (GCNN) [23] and conditional random field (CRF) [26] for breast cancer treatment entity normalization in Chinese clinical text, which fully takes advantage of the contextual information mainly in Chinese and sequential interactive information. Specifically, the main contributions of our work can be summarized as follows:

- We propose PASCAL, an end-to-end, accurate and efficient framework with GCNN and CRF to normalize breast cancer treatment, which fully makes use of the sequential interactive information and implicit context information in Chinese clinical text. To the best of our knowledge, this is the first work to introduce GCNN and CRF specialized for TEN. Moreover, the experiments on a large real-world breast cancer EHR dataset illustrate the effectiveness and efficiency of the framework.
- In the pseudo cascade structure, we incorporate TER into the framework as an auxiliary task to propagate useful implicit information and assist in optimizing the shared parameters. The final experimental results prove the necessity of the auxiliary recognition task.
- We present a biased loss function with an adjustable parameter *γ* to strategically optimize the parameters and seek an optimized balance between the contributions of assistant optimization and providing information.

## Materials and problem definition

Chinese medical named entity normalization (TEN) aims to map different medical terms from Chinese clinical text, as shown in Table 1, onto a controlled vocabulary, which can be regarded as a multiclass learning task. Nevertheless, the ambiguity in the boundary of Chinese words can cause segmentation errors, which could introduce noise into the downstream task. Considering this, we label the sequence at the character level to mitigate the error transmission. In addition, we incorporate an auxiliary task TER to further assist in regulating the parameters from shared layers. Next, we introduce the input and output of the TEN task and describe the primary definitions of the problem.

**Table 1:** An illustration example of TEN in a clinical text

Clinical text	Real text:										
	
	Translated text: After admission, the relevant examination was improved, without chemotherapy										
	contraindications, and **EC sequential TH regimen** was given to assist chemotherapy. The patient										
	reported nausea, vomiting and dysphagia, and provided Dolasetron mesylate injection. After										
	chemotherapy, there was no obvious side effect, and life sign was stable;										
Characters sequence	...		E	C			T	H			...
Standard entity	...	O	EC-TH	EC-TH	EC-TH	EC-TH	EC-TH	EC-TH	O	O	...

### Input and output data

Owing to the complexity of the real-world database, we extract the clinical notes from EHRs. Let **D** = {**p**_1_,**p**_2_,...,**p**_*n*_} denote the patients from the EHR. **p**_*i*_ = {**v**_1_,**v**_2_,...,**v**_*k*_} is the *i*-th patient, where **v**_*k*_ denotes a visit encounter and *k* is the number of visits for the patient. For a visit **v**_*k*_, it might generate multiple treatment records {**X**_1_,**X**_2_,...,**X**_*l*_} for the therapy of breast cancer, where *l* represents the number of treatments in a visit. We treat the records as different input sequences. As shown in Table 1, the input clinical text **X**_*l*_ it contains multiple characters {*x*_1_,*x*_2_,...,*x*_*N*_}, where *N* denotes the number of characters in a sequence. The labels, namely, standard entities, are from the standard treatment regimens database **C** = {**r**_1_,**r**_2_,...,**r**_*j*_}, where *r*_*j*_ is an entity and *j* is the number of entities.

### Problem definition

The Chinese EHRs contain various mentions about the same entity because the data can come from various hospitals and be recorded by different doctors under their own guidelines. Therefore, the aim of TEN is to map the mention with a nonstandard name to a specified controlled vocabulary from the treatment regimens database **R**:

1 $$ \left(y_{1},y_{2},...,y_{N}\right) = f\left(x_{1}, x_{2},..., x_{N}\right)   $$

where *y*_1_,*y*_2_,...,*y*_*N*_∈**R** is the normalized entity from the treatment regimens database, *x*_1_,*x*_2_,...,*x*_*N*_ is the input characters from a clinical sentence, and N is the number of characters in one clinical sentence **X**_*l*_. In this one-vs-one method (character-vs-label), we can not only ensure the correctness of normalization but also understand the location of the treatment entity.

## Methods

**Fig. 2:**
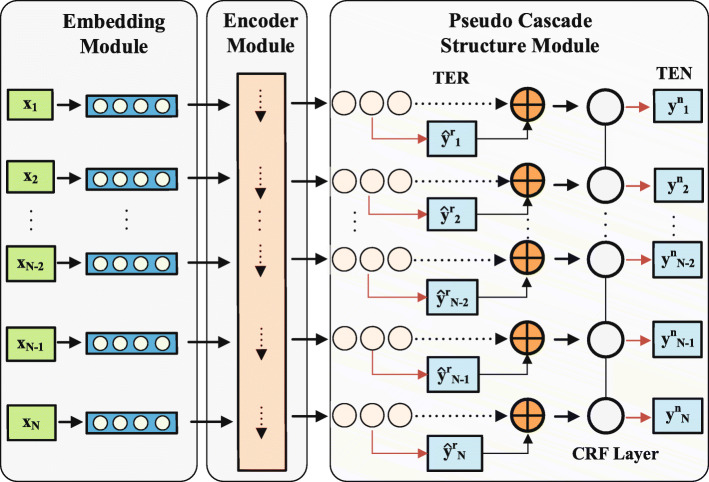
Main architecture of PASCAL model. PASCAL consists of four modules: character embedding module, encoder module (containing a gated convolutional neural network to learn the shared representation with temporal relationship), pseudo cascade structure module (including the enhanced primary task TEN and an auxiliary task TER)

In this section, we present a pseudo cascade learning framework with gated convolutional networks and a conditional random field to address the TEN task. As shown in Fig. 2, the model is composed of four key modules: embedding layer, GCNN encoder module, pseudo cascade structure, and the CRF layer. First, the embedding layer projects the Chinese characters into dense vector representations. Then, the representations are fed into the encoder GCNN to capture the contextual relationships and long-term dependencies by the convolutional network and gating mechanism. After obtaining the contextual features, a pseudo cascade structure, which includes a softmax layer, an auxiliary TER layer and an information fusion layer, is utilized to obtain the fused information vector representation. Finally, to obtain more accurate normalization outcomes, we deploy a CRF layer due to its superiority in capturing the internal and contextual relationships within labels. Subsequent sections detail the components of the pseudo cascade learning framework (PASCAL).

### Embedding layer

As discussed in “Materials and problem definition” section, Chinese sentences have their nature without separators between words, and word segmentation is usually treated as the first step for clinical test mining. Word segmentation can cause ambiguity in the boundaries of Chinese words. To address the above problems, our proposed PASCAL is based on the character level input to avoid introducing noise caused by segmentation errors. Formally, as shown in Table 1, given a clinical treatment sentence **X**_*l*_ = {*x*_1_,*x*_2_,...,*x*_*N*_}, The model first maps the characters to dense embedding representations. Specifically, the character embedding $\boldsymbol {e}_{i} \in \mathbb {R}^{d_{e}}$ is extracted from embedding matrix $\boldsymbol {W}_{e} \in \mathbb {R}^{|N| \times d_{e}}$ that can be learned for every character *x*_*i*_, where *i*∈{1,2,...,*N*} and *d*_*e*_ is a hyperparameter denoting the embedding size. Then, the character embedding vectors can be treated as a sequence that is fed into the encoder to mine more complex relations.

### Gated convolutional neural network module

**Fig. 3:**
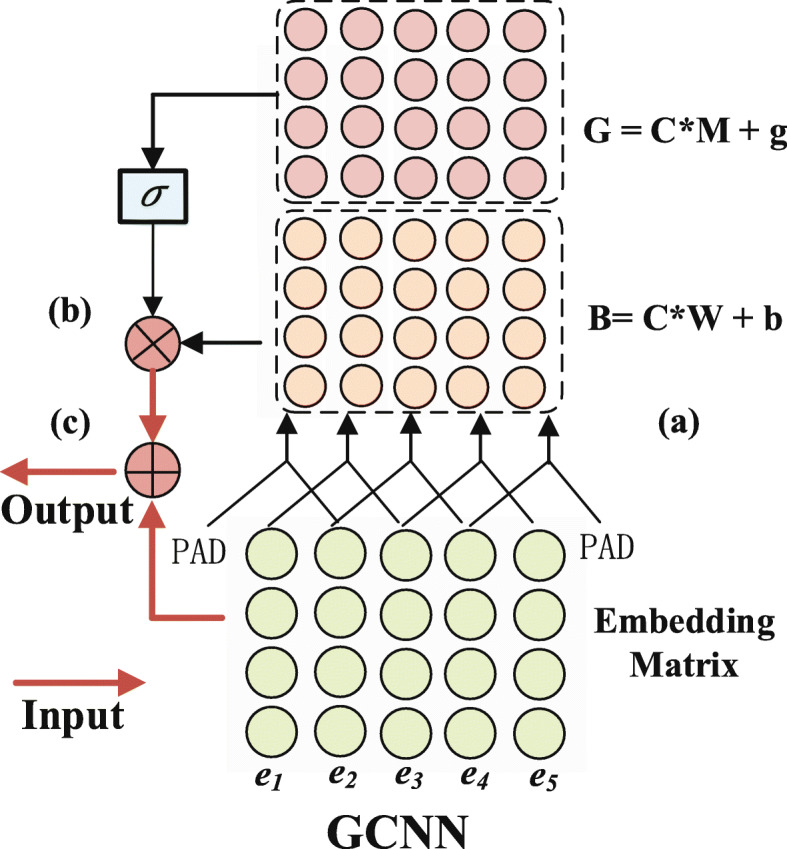
Detailed structure of encoder module: gated convolutional neural network (GCNN). GCNN consists of three key parts: convolutional block, gating block and residual connection

As shown in Fig. 2, the gated convolutional neural network (GCNN) is selected as the encoder of PASCAL, and the detailed substructures are shown in Fig. 3. In the figure, GCNN consists of three blocks, including a convolutional block, a gating block and a residual connection, which enable the GCNN to capture the contextual relationships and long-term dependencies in an efficient manner.

As shown in Fig. 3a, the input to the convolutional block is a sequence of character embeddings **C**={***e***_1_,***e***_2_,...,***e***_*N*_}, where $\mathbf {C}\in \mathbb {R}^{|N| \times d_{e}}$, |*N*| is the number of characters, and *d*_*e*_ is the embedding size. Then, the matrix **C** is sent to the one-dimensional convolutional neural network, and finally, we obtain the outputs **B**=**C**∗**W**+**b** and **G**=**C**∗**M**+**g**, where **W**, $\mathbf {M} \in \mathbb {R}^{k \times d_{e} \times d_{h}}$, $\mathbf {b} \in \mathbb {R}^{d_{h}}$ and $\mathbf {g} \in \mathbb {R}^{d_{h}}$ are the parameters to be learned. Furthermore, *d*_*h*_ denotes the output dimension, and *k* denotes the patch size in the convolutional process.

Following the convolutional operation is the gating block, as shown in Fig. 3b, in which a gated linear unit (GLU) [23] is utilized to control the information flows by selecting features through a sigmoid activation function:

2 $$ \mathbf{h}_{l}\left(\mathbf{C}\right) =\mathbf{B} \odot \sigma\left(\mathbf{G}\right),   $$

where **h**_*l*_ is the output of one hidden layer. ⊙ is the elementwise product between matrices, and *σ* is the sigmoid activation function.

Finally, considering the computational efficiency, a residual connection [27] is further added to the block, which means that the final output consists of two parts, the output of GLU and the input of the block. Thus, **C**+**h**_*l*_(**C**) is the final output of the *l*-th layer.

### Pseudo cascade structure

One limitation of pipeline approaches is that the errors from TER propagate to subsequent TEN tasks. Therefore, we present the pseudo cascade learning structure that can mitigate the adverse impact and enhance the positive effect. As shown in [28], the auxiliary tasks can be regarded as a kind of regularization to boost the performance of the main tasks. In addition, [29] adds unsupervised auxiliary tasks to improve the outcomes of emotional attributes. Likewise, we leverage the auxiliary task as an additional regularization to assist the primary tasks, both of which constitute the pseudo cascade learning structure. The detailed architecture is described as follows.

First, the encoder GCNN generates informative feature vectors with contextual relationships and long-term dependencies. Then, as shown in Fig. 2, they are further fed into the pseudo cascade structure to fulfill two tasks: Chinese medical named entity recognition (TER, an auxiliary task) and Chinese medical named entity normalization (TEN, the primary task). Although the TER task is assistant, it is indispensable for the regularization of shared parameters and the transmission of useful information. In addition, the pseudo cascade structure also includes the softmax activation layers and the critical CRF layer.

#### Auxiliary task: TER

In the auxiliary task TER, to recognize the medical entities ${y}^{r}_{1}, {y}^{r}_{2},..., {y}^{r}_{i}$, we take the informative feature vectors **H** = {**h**_1_,**h**_2_,...,**h**_*N*_} from the encoder GCNN as the input. With the help of a linear layer and a softmax layer, we can obtain the recognized entity:

3 $$ \hat{\textbf{y}}^{r}_{i} = \operatorname{softmax}\left(\mathbf{W}^{r} \mathbf{h}_{i}+\mathbf{b}^{r}\right),   $$

where $\hat {\textbf {y}}^{r}_{i}$ is the recognized entity, $\mathbf {W}^{r} \in \mathbb {R}^{d_{r} \times d_{h}}$, $\mathbf {b}^{r}\in \mathbb {R}^{d_{r}}$ are the learned parameters, and $\mathbf {h}_{i}\in \mathbb {R}^{d_{h}}$ is the *i*-th input vector. $\hat {\textbf {y}}^{r}_{i}$ is regarded as additional information to be transmitted to the primary task.

#### Primary task: enhanced TEN

As mentioned above, in the primary task, we not only leverage the information from the encoder GCNN, **H** = {**h**_1_,**h**_2_,...,**h**_*N*_} but also utilize the information from the auxiliary TER task. To be more specific, we directly take advantage of the concatenation method to integrate them:

4 $$ \textbf{h}^{c}_{i} = \left[\mathbf{h}_{i}, \hat{\textbf{y}}^{r}_{i}\right].   $$

where $\textbf {h}^{c}_{i}$ denotes the input of the next CRF layer, $\hat {\textbf {y}}^{r}_{i}$ is the recognized entity, **h**_*i*_ is the output of the encoder GCNN and $\hat {\textbf {y}}^{r}_{i}$ is the predicted outcome from the auxiliary TER task. Therefore, the input of the CRF layer can be defined as **H**^*c*^ = $\left \{\textbf {h}^{c}_{1}, \textbf {h}^{c}_{2},..., \textbf {h}^{c}_{N} \right \}$.

**CRF layer** To better utilize the contextual information and obtain the optimum global path, we leverage CRF [26] to model the normalization sequence and predict the TEN sequential results.

The label sequence of characters is denoted as **Y**=[**y**_1_,**y**_2_,…,**y**_*N*_], where $\textbf {y}_{i} \in \mathbb {R}^{|C|}$ is the *i*-th character’s label with one-hot representation and |*C*| is the number of treatment regimens in the database. The input of the CRF layer is the integrated representation, namely, **H**^*c*^ = $\left \{\textbf {h}^{c}_{1}, \textbf {h}^{c}_{2},..., \textbf {h}^{c}_{N} \right \}$. Moreover, the CRF is a probabilistic model, and the conditional probability of **Y** given input **H**^*c*^ is calculated as follows:

5 $$ p\left(\mathbf{Y} | \textbf{H}^{c}; \theta\right)=\frac{\prod\nolimits_{i=1}^{N} \psi\left(\mathbf{h}^{c}_{i}, \textbf{y}_{i}, \textbf{y}_{i-1}\right)}{{\sum\nolimits}_{\mathbf{y}^{\prime} \in \mathcal{Y}(s)} \prod\nolimits_{i=1}^{N} \psi\left(\mathbf{h}^{c}_{i}, {\textbf{y}_{i}}^{\prime}, {\textbf{y}_{i-1}}^{\prime}\right)},   $$

where $\mathcal {Y}(s)$ denotes the set of all possible label sequences under a given sentence, *θ* denotes the learned parameters, and $\psi \left (\mathbf {h}^{c}_{i}, \textbf {y}^{n}_{i}, \textbf {y}^{n}_{i-1}\right)$ denotes the potential function:

6 $$ \psi\left(\mathbf{h}^{c}_{i}, \textbf{y}_{i}, \textbf{y}_{i-1}\right) = \exp \left(\textbf{y}^{T}_{i} \mathbf{W}^{T} \mathbf{h}^{c}_{i} + \textbf{y}^{T}_{i} \mathbf{T} \textbf{y}_{i-1}\right),   $$

where $\mathbf {W} \in \mathbb {R}^{|d_{r}+d_{h}|\times |C|}$ and $\mathbf {T} \in \mathbb {R}^{|C|\times |C|}$ are the learned parameters, both of which constitute *θ* in Eq. (5).

### Biased loss function

To enhance the performance of TEN, we present a biased loss function for the pseudo cascade learning framework, which can partially influence the optimization process by adjusting the proportion of TEN loss and TER loss.

#### TER loss

For auxiliary task TER, we employ the binary cross-entropy between the ground truth label $\textbf {y}^{r}_{i}$ and the predicted $\hat {\textbf {y}}^{r}_{i}$ as the objective loss function:

7 $$ \mathcal{L}_{TER} = -\sum\limits_{i=1}^{N}\left(\textbf{y}^{r}_{i} \log \hat{\textbf{y}}^{r}_{i}+\left(1-\textbf{y}^{r}_{i}\right) \log \left(1-\hat{\textbf{y}}^{r}_{i}\right)\right).   $$

#### TEN loss

For the enhanced TEN task, we adopt the negative log-likelihood over all training samples as the loss function of CRF, which can be computed as follows:

8 $$ \mathcal{L}_{TEN}=-\sum\limits_{s \in \mathcal{D}} \log \left(p\left(\mathbf{Y}_{s} | \textbf{H}_{s}^{c}; \theta \right) \right)   $$

where $\mathcal {D}$ is the set of medical sentences of training data, *s* denotes one sequential sentence in $\mathcal {D}$, **Y**_*s*_ is the label sequence and $\textbf {H}_{s}^{c}$ is the integrated input representation.

#### Biased loss function

To strategically optimize the model parameters, we incorporate a static parameter *γ*, which can be called a bias parameter, into the biased loss function for indirectly tuning the optimization process. The biased loss function is:

9 $$ \mathcal{L}_{BL} = \gamma\times\mathcal{L}_{TEN} + \left(1-\gamma\right)\times\mathcal{L}_{TER},   $$

where 0<*γ*<1 and $\mathcal {L}_{BL}$ is the combined loss function. Furthermore, to obtain the best model, we should find a balance between $\mathcal {L}_{TEN}$ and $\mathcal {L}_{TER}$ by fine tuning the bias parameter *γ*. The detailed information is discussed in “Bias parameter analysis” section.

## Experiments

### Data

To show the effectiveness of PASCAL, we evaluated it on a real-world EHR dataset containing 12,700 clinical records from Chinese third grade and class-A hospitals. As introduced in Fig. 1, treatment regimens, from the clinical text with a detailed description, might be recorded by different doctors following their own guidelines, which can generate nonstandardized terms on the clinical records. Hence, our objective is to map the treatment regimens onto the controlled vocabulary from the latest *GUIDELINES* [9] (the authoritative reference for breast cancer physicians in China). For each patient, we extracted the clinical treatment regimens from their electronic health records and integrated them. As the length of nearly 99% clinical texts in the datasets is less than 256, in this paper, we employ clinical texts whose length is less than 256 in the following experiments. To maintain relative independence, we partition the records into training data and test data by a ratio of 8:2 based on the patients. Therefore, it contains 209,677 sentences for training and 52,420 sentences for testing. In the experiment, the training data are randomly sampled at 10% for validation, and the remaining data are used for training.

### Settings and hyperparameters

To evaluate the effectiveness of framework PASCAL and the influence of each key component, we design various experiments on a real-world database. First, we choose the three latest sequential models as baselines, including Bi-LSTM [24], bidirectional OnLSTM [30] and TCN [22], to obtain an accuracy comparison with GCNN. We also conduct experiments for the single task to compare CRF with softmax in a sequential multiclass classification task. In addition, to further evaluate the performance of our model, one state-of-the-art multitask learning model, we call it feedback [17], is used as another baseline model in the experiment. Finally, we dynamically adjust the values of *γ* to realize the best model performance and to validate the impact of the bias parameter on model performance via experiments. Moreover, it is worth noting that most experiments are conducted based on univariate analysis.

To achieve the optimal normalization results, the hyperparameters are set as follows: the dimension of character embedding is set as 200, the number of filters in the first convolutional layer is set as 128 and in the following three connected layers is set as 256, the size of convolutional kernels in the CNN layer is set as 3, the number of convolutional layers is 4, the number of residual blocks is 3, the dropout probability is set as 0.5, the learning rate is set as 0.001 and the batch size is set as 256. We select the hyperparameters in terms of cross-validation on training data and choose the average result of 10 experiments as the result. In addition, the parameters are initialized with Xavier initialization, and we take the LazyAdam [31] optimizer for all neural networks. Finally, we employ the Keras library [32] with the TensorFlow [33] backend, and all models are run on a single NVIDIA Tesla P40.

### Evaluation metrics

To fully evaluate the proposed approaches, we use three prevalent evaluation metrics to provide a comparison among different approaches. The metrics in [34] are precision, recall, and the F1-measure:

10 $$\begin{array}{*{20}l} { Precision} =\frac{T P}{T P+F P} \end{array} $$

11 $$\begin{array}{*{20}l} { Recall }=\frac{T P}{T P+F N} \end{array} $$

12 $$\begin{array}{*{20}l} { F1-Measure }=\frac{2 \times \text { Precision} \times \text { Recall }}{\text { Precision }+\text { Recall }} \end{array} $$

where *FP* and *TP* are the number of false positives and true positives, respectively.

## Results

### Performance comparison

Table 2 illustrates the performance comparison between baselines and our proposed approach concerning three evaluation metrics on a real-world breast cancer dataset for treatment entity normalization (TEN) in Chinese clinical text. *Softmax* and *CRF* denote the softmax layer and CRF layer for the single task of normalization, respectively. Moreover, *PASCAL (Softmax + CRF)* denotes our proposed cascade learning framework with a softmax layer for the auxiliary task and a CRF layer for the primary task.

**Table 2:** Performance comparison on a real-world breast cancer dataset

Model	Precision	Recall	F1
Softmax			
Bi-LSTM	0.8171±0.0143	0.8796±0.0264	0.8472±0.0221
Bi-OnLSTM	0.8316±0.0205	0.8978±0.0139	0.8635±0.0152
TCN	0.7135±0.0129	0.8218±0.0231	0.7638±0.0245
GCNN	0.8817±0.0117	0.9016±0.0210	0.8921±0.0124
CRF			
Bi-LSTM	0.8682±0.0125	0.8905±0.0238	0.8792±0.0201
Bi-OnLSTM	0.8678±0.0187	0.8952±0.0145	0.8813±0.0168
TCN	0.8486±0.0089	0.9076±0.0214	0.8771±0.0179
GCNN	**0.9443**±0.0126	0.9628±0.0181	0.9535±0.0094
PASCAL (Softmax + CRF)			
Bi-LSTM	0.8931±0.0153	0.9121±0.0183	0.9025±0.0168
Bi-OnLSTM	0.9078±0.0149	0.9348±0.0156	0.9211±0.0175
TCN	0.8744±0.0102	0.9342±0.0192	0.9033±0.0149
GCNN	0.9413±0.0156	**0.9770**±0.0147	**0.9589**±0.0054

As seen in Table 2, our proposed framework outperforms all the baselines on precision, recall and F1. Specifically, for our proposed framework with encoder TCN, we observe that the F1 score exceeds approximately 13.9% and 2.62%, the recall score exceeds approximately 11.2% and 2.66%, and the precision score exceeds approximately 16.1% and 2.6% compared to that of *softmax* and *CRF*, respectively. This means that our proposed pseudo cascade learning framework can fully take advantage of the auxiliary TER task to optimize the shared parameters and propagate the implicit information to the primary TEN task. Moreover, for *PASCAL* with encoder GCNN, the F1 score and recall score outperform others except for precision. This phenomenon shows that *PASCAL* is more inclined to the correctness of normalized regimens but neglects part of the ground truth regimens. However, the recall and F1 metrics are more meaningful than the precision metric in health informatics.

Concerning the critical encoder, as shown in Table 2, GCNN performs better than other encoders on all evaluation metrics under the same framework. This partly indicates that GCNN has a stronger ability to capture long-range dependencies and mine the contextual relationships via the convolutional blocks and gating block. In addition, comparing *CRF* with *softmax*, we observe that the former with the CRF layer obtains higher performance than the latter with the softmax layer. The reason is that the neighboring TEN labels have strong dependencies that can be captured by CRF.

Another meaningful finding is that the models with GCNN perform much better than the model with Bi-OnLSTM. Both models can utilize hierarchical information to obtain better performance. However, the difference is that the latter integrates the intrinsic tree structures into RNN to obtain ordered neurons, while the former builds the hierarchical structure via stacked CNN layers to capture local and long-range dependencies and introduces a gating block to avoid gradient vanishing problems.

**Fig. 4:**
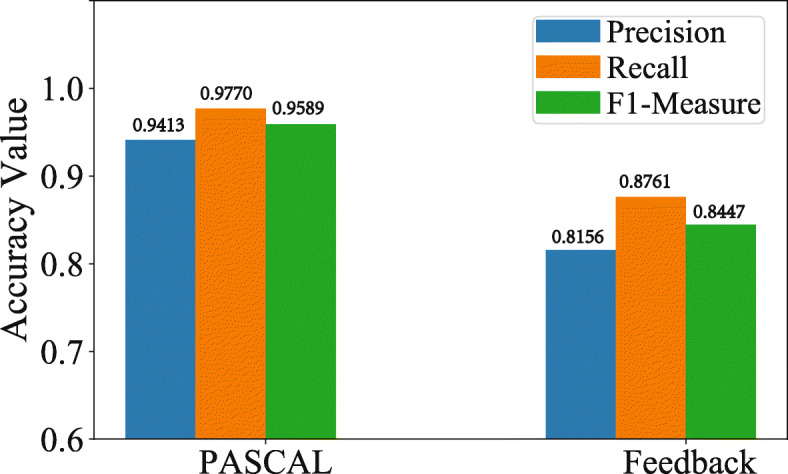
Accuracy comparison between PASCAL and Feedback [17]

Furthermore, as shown in Fig. 4, the performance of PASCAL obviously outperforms Feedback [17] with respect to three evaluation metrics. We think there are three main reasons for this. First, the explicit feedback approach is designed for medical entity recognition and normalization in English clinical text, while the PASCAL model is developed for the TEN task in Chinese clinical text. Second, the constituent characters in Chinese clinical text are complicated and not only contain Chinese characters but also mix English characters. The relations between them are intricate and varied. The powerful blocks of encoder GCNN enable PASCAL to better capture the contextual relationships and long-term dependencies in clinical sentences. Third, the pseudo cascade structure in PASCAL can further improve the model performance by retaining useful information and mitigating error propagation. In addition, the incorporation of CRF can better utilize contextual information to normalize the treatment entity. Therefore, based on the above analysis, our model with GCNN and CRF is the most suitable approach for the TEN task for breast cancer.

### Computational efficiency

The aforementioned analyses mainly concentrate on the aspect of normalization accuracy. However, it is well known that computational efficiency is a critical factor in industrial applications. The main reason is that the computational efficiency within finite computational ability is much more important than a slight improvement in accuracy under some circumstances. For instance, in mobile health monitoring, the responsive time of the device has a great influence on the popularization rate. From the perspective of clinical doctors, what they need is saving their time for decision-making and not wasting their time on it. Thus, we must maintain a balance between efficiency and accuracy when choosing the approaches.

**Fig. 5:**
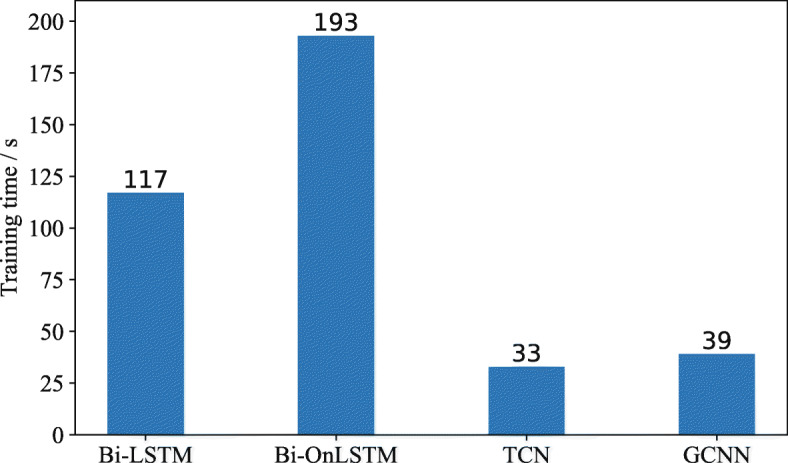
Computational efficiency comparison of *PASCAL* with different encoders

As shown in Fig. 5, our presented PASCAL framework with different encoders spends different training times finishing one epoch. We find that Bi-OnLSTM spends 193*s* on one training epoch, Bi-LSTM needs 117*s*, while TCN and GCNN need 33*s* and 39*s*, respectively. The reason lies in the different operating mechanisms between recurrent networks and convolutional networks. The recurrent network-based models, such as Bi-LSTM, cannot be parallelized over the characters of a sentence because the next outputs rely on the previous state. However, convolutional networks are very amenable to parallel computing because the computation of all input characters in a sentence can be performed simultaneously. Moreover, the training efficiency of the TCN is higher than that of the GCNN because it directly imposes temporal information on the convolutional process and does not rely on the gating block, which slightly improves the efficiency. However, the performance of GCNN on Precision, Recall and F1 is 6.7%, 4.3% and 5.6% higher than TCN. Therefore, after comprehensively considering the accuracy and efficiency, we choose GCNN as the encoder of the pseudo cascade learning framework.

### Bias parameter analysis

The main task of PASCAL is to normalize the treatment entity into standard vocabulary with the help of an auxiliary TEN task. *γ* represents the proportion of TEN loss in the training process, and (1−*γ*) denotes the proportion of TER loss. Considering TEN as the primary task, we manually adjust the proportion of *γ* in the biased loss function $\mathcal {L}_{BL}$ from 0.5 to 0.9 to explore the influence of *γ* on the normalization performance. Table 3 shows that as the value of *γ* increases, the normalization accuracy also increases, which indicates that the optimization process is gradually inclined to the orientation that is beneficial to the TEN task. We observe that the improvement process becomes unstable with the increase in the *γ* value. For instance, the recall score when *γ*=0.7 is lower than *γ*=0.6. We hypothesize that the main reason is that the increase in *γ* means a decrease in 1−*γ*, which indirectly influences the optimization process related to the TER auxiliary task. Moreover, the affected auxiliary TER will further influence the optimization process of shared parameters. Therefore, we should rationally select the appropriate value of *γ* in practical applications.

**Table 3:** Performance comparison with regard to different bias values

*γ*	Precision	Recall	F1
0.5	0.9390	0.9769	0.9576
0.6	0.9347	0.9773	0.9555
0.7	0.9413	0.9770	0.9589
0.8	0.9402	0.9779	0.9587
0.9	**0.9435**	**0.9780**	**0.9604**

## Error analysis

Table 4 exhibits four general errors in different categories obtained from the testing results. The displayed breast cancer treatments are extracted from complicated clinical text (Fig. 1b) and concatenated with the entity positions. Specifically, the table lists the normalization results and corresponding labels for each error case. For instance, [’AC’, 17, 19], ’AC’ denotes the treatment regimen of breast cancer, 17 denotes the starting index of the entity in a sentence, and 19 denotes the ending index. Only when the entity and the starting and ending indexes are all accurate can the normalized results be recognized as correct. In error case 1, there is an extra normalized entity [^′^*AC*−*T*^′^,11,14], which is regarded as a correct normalization result. This occurs because the entity label is missing in the sentence, which can be an inevitable real case in the dataset with artificial labels. However, the error case also confirms the normalization effectiveness of our method. Error case 2 belongs to the general normalization mistakes via our methods. However, for error case 3, it is difficult to normalize, especially when the treatment regimens rarely exist in the training set. In that case, the algorithm mapped the regimen onto the most similar normalization entity. Likewise, in error case 4, the normalized indexes deviate from the standard position, which brings about another unnecessary entity ’EC-T’ that is an error due to the high similarity to ’FEC-T’. All of the above-discussed error cases will be further solved in our future work and practical applications.

**Table 4:** Error cases about the breast cancer treatment normalization

		

Notes: The treatments are specifically extracted from the clinical context that describes the treatment process of the patient. Treatments in red color indicates the error cases on both the name and position of treatment

## Conclusion and outlook

In this paper, we present a novel pseudo cascade learning framework with a gated convolutional neural network and conditional random field, named PASCAL, for breast cancer entity normalization. Unlike traditional LSTM-based models, our approaches improve the ability to capture the local and long-range dependencies in a sentence by a gated convolutional network (GCNN) and enhance the training efficiency. We design a pseudo cascade structure with an auxiliary TER task to provide auxiliary assistance for optimizing the shared parameters and propagating the useful information and with a biased loss function to further optimize the TEN process. Moreover, we employ a conditional random field (CRF) to obtain the optimized normalization results by considering the previous labels and contextual information. Finally, we conduct extensive experiments on a real-world dataset of treatment regimens for breast cancer, and the experimental results validate the effectiveness and efficiency of our proposed approaches. In general, the presented methods can be utilized to solve the Chinese named entity normalization in any other field.

We further improve the performance from the following three aspects. First, we attempt to utilize the public corpus to pretrain the character embedding for better performance. Second, we integrate the domain knowledge about breast cancer into the model to enable the model to be more targeted. Third, we consider dynamically adjusting the optimization process by replacing static *γ* with a dynamic parameter that can be learned from the neural networks. Finally, we leveraged the normalized treatment and clinical laboratory measurements to recommend breast cancer treatment for patients.

## Data Availability

The breast cancer dataset that supports our research in the paper is not available since there are many privacy information in the clinical text, and no related act can be referred for medical data publication in China. In addition, the employed data belong to in-hospital desensitization text data, not involving any patient privacy information, and only for scientific research.

## Abbreviations

Bi-LSTM: Bidirectional long short-term memory
CRF: Conditional random field
EHR: Electronic health record
GCNN: Gated convolutional neural network
TEN: Treatment entity normalization
TER: Treatment entity recognition
PASCAL: Pseudo cascade learning
